# Non-invasive prediction of EGFR gene mutations in non-small cell lung cancer by multi-parameter CT perfusion imaging

**DOI:** 10.3389/fmed.2025.1660923

**Published:** 2025-10-03

**Authors:** Can Chen, Xiao Liu, Anlvna Li, Xiongjian Zhang, Qianyi Xie, Rui Guo, Wei Li, Qi Liang, Xiaoping Tang

**Affiliations:** ^1^Department of Radiology, The Second Affiliated Hospital, Jiangxi Medical College, Nanchang University, Nanchang, China; ^2^Department of Radiology, The Third Xiangya Hospital, Central South University, Changsha, China; ^3^Intelligent Medical Imaging of Jiangxi Key Laboratory, Nanchang, China

**Keywords:** non-invasive, CT perfusion imaging, non-small cell lung cancer, epidermal growthfactor receptor, gene mutation

## Abstract

**Background and objectives:**

In current clinical practice, invasive methods such as biopsy are commonly used to obtain tumor tissues for epidermal growth factor receptor (EGFR) mutation detection in patients with non-small cell lung cancer (NSCLC). This study aimed to explore the underlying association between various quantitative parameters of CT perfusion imaging (CTPI) and EGFR mutation, thus providing a new auxiliary diagnosis basis for non-invasive prediction of EGFR mutation status in patients with NSCLC.

**Methods:**

Patients with a confirmed NSCLC diagnosis by surgery or biopsy were prospectively enrolled. All patients underwent pulmonary CTPI within 1 week before biopsy, as well as EGFR gene detection after biopsy, and were then divided into the EGFR mutation group and the wild-type group. Differences in quantitative parameters between the two groups were analyzed, and significant variables were identified for further construction of the predictive model. The receiver operating characteristic (ROC) curves were constructed, and the area under curve (AUC) was calculated to assess the predictive performance.

**Results:**

A total of 86 patients were included, including 45 women and 41 men. There were 47 cases in the mutation group and 39 cases in the wild-type group. A univariate analysis showed that compared with the wild-type group, blood volume (BV) (5.56 ± 1.51 vs. 3.04 ± 1.07, *p* < 0.001), time to peak (TTP) (29.31 ± 5.12 vs. 25.99 ± 5.68, *p* = 0.006), and permeability surface (PS) (18.98 ± 6.79 vs. 11.77 ± 5.56, *p* < 0.001) were all higher in the mutation group. No statistical differences were found in the other five quantitative parameters (*p* > 0.05). A multivariate logistic regression analysis identified BV (*p* < 0.001), TTP (*p* = 0.029), and PS (*p* = 0.014) as independent predictors of EGFR mutation. According to the ROC, the AUC of BV, TTP, and PS were 0.916, 0.739, and 0.788, respectively, and the corresponding cut-off values were 4.69, 23.84, and 12.11, respectively. The AUC of the combined predictive model (BV + TTP + PS) reached 0.956, which was superior to that of any single parameter (*p* < 0.05).

**Conclusion:**

BV, TTP, and PS were independent predictors of EGFR mutation in patients with NSCLC. The combined CTPI parameter model (BV + TTP + PS) had the highest predictive performance and could be more reliable than any single parameter in clinical auxiliary diagnosis.

## 1 Introduction

Cancer is a major public health problem worldwide, and its morbidity and mortality are increasing year by year. According to data released by the World Health Organization International Agency for Research on Cancer (IARC) in 2024, lung cancer remains the most common cancer in the world and the leading cause of death from cancer (1). In China, lung cancer is the malignant tumor with the highest morbidity and mortality in both men and women, with 1,066,060 cases of lung cancer and 733,000 deaths in 2022 (2). Lung cancer can be divided into non-small cell lung cancer (NSCLC) and small cell lung cancer (SCLC) according to histopathology, of which NSCLC accounts for more than 85% (3, 4), and its main types include adenocarcinoma, squamous cell carcinoma, and large cell lung cancer, of which adenocarcinoma is the most common subtype.

Numerous studies have found that approximately 14 and 30% of patients with NSCLC in Europe and Asia present with epidermal growth factor receptor (EGFR) gene mutations, respectively (5, 6). It is worth noting that the proportion of EGFR mutations among Chinese patients with NSCLC is as high as approximately 55.9% (7). Recently, with the discovery of a series of driver genes of NSCLC, especially the most common EGFR gene, molecular targeted therapy has resulted in a significant survival benefit for patients with advanced NSCLC (8). On the other hand, for patients with NSCLC lacking driver mutations, although they may receive immunotherapy or a combination of radiotherapy and chemotherapy, their survival rate remains very low (9). According to molecular testing guidelines for patients with lung cancer, molecular biological tests such as EGFR mutation, anaplastic lymphoma kinase (ALK), and c-ros oncogene 1 (ROS1) rearrangement should be routinely performed for NSCLC containing adenocarcinoma components, regardless of its clinical characteristics (such as smoking history, gender, race, etc.), of which EGFR is a mandatory gene for NSCLC (strong recommendation) (10, 11). Histological examination serves as the gold standard for EGFR mutation detection, while tumor tissue can only be obtained by puncture or fiberoptic bronchoscopic biopsy in inoperable patients with advanced lung cancer, of which CT-guided lung biopsy is the preferred method (12). In real-world clinical practice, biopsy has many limitations. First, some patients refuse or have contraindications to needle biopsy due to its invasive feature. Second, the examination might become difficult because of tumor size and location, resulting in obtaining limited biopsy tissue samples and poor detection effect. Third, multiple needle biopsies will aggravate the physical, mental, and economic burden of patients due its high price. Therefore, it is necessary to explore a non-invasive and simple method to predict EGFR gene mutation in cases with NSCLC, which can provide a reference for appropriate targeted therapy decisions when biopsy specimens are not available. This study aims to explore the underlying association between various quantitative parameters of CT perfusion imaging (CTPI) and EGFR mutation, as well as to identify independent predictors of EGFR mutations and to construct a combined model with good predictive performance.

## 2 Materials and methods

### 2.1 Study population

**Figure 1:**
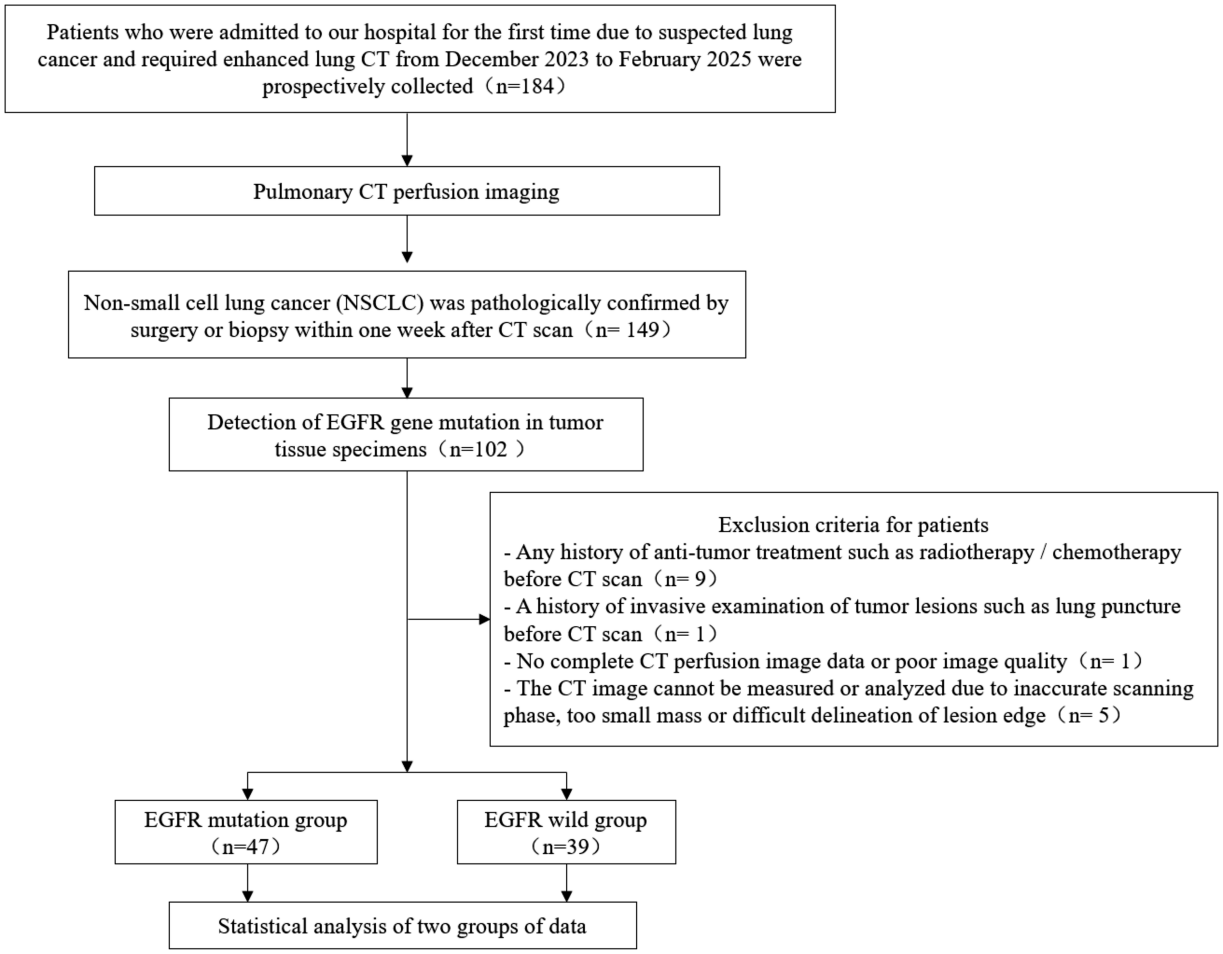
Flowchart of patient enrollment.

Patients who required enhanced CT due to suspected lung cancer in our hospital were prospectively recruited from December 2023 to February 2025. The inclusion criteria were as follows: (1) patients received CTPI examination in our hospital; (2) pathological NSCLC diagnosis confirmed by surgery or biopsy; (3) EGFR gene mutation detection was performed on tumor tissues; and (4) an interval not exceeding 1 week between CTPI examination and tumor tissue biopsy. Patients were excluded if (1) they had history of any anti-tumor treatment, such as radiotherapy or chemotherapy, before CTPI examination; (2) they had history of traumatic examination of tumor lesions such as puncture before CTPI examination; (3) the image data of CTPI was incomplete or had poor quality; (4) the image data of CTPI could not be measured and analyzed due to inaccurate scanning phase, too small mass, or blurred tumor outline. The flow diagram of patient enrollment is shown in Figure 1.

The study was conducted in accordance with the Declaration of Helsinki and was approved by the Institutional Review Board of The Third Xiangya Hospital, Central South University (No.2022-S116). Informed consents for the CTPI examination and the utilization of genetic testing results were obtained from all participants.

### 2.2 Pulmonary CTPI procedure

#### 2.2.1 Preparation

Patients without contrast-enhanced CT contraindications were required to fast for 4–6 h before the examination. Patients received breathing training prior to CTPI and were instructed to remain motionless during the scan to reduce the interference of breathing or motion artifacts on the later image processing.

#### 2.2.2 CTPI instrument and technical parameters

All patients received perfusion scan using 256-slice Revolution CT (GE Healthcare, Waukesha, WI, USA). The location of the target lesion was determined on the lung localization image or non-enhanced images so that the perfusion scope covered the whole target lesion. Perfusion scanning and reconstruction parameters were as follows: scan mode = axial scan, detector width (scanning range) = 10 cm, tube voltage = 100 kV, tube current = 120 mA (manual mode), tube rotation time = 0.5 s, acquisition slice thickness = 0.625 mm, reconstruction slice thickness = 5 mm, iteration weight: ASiR-V = 50%. A total of 50 mL iodine contrast agent (350 mg I/ml, Starry Pharmaceutical Co., Ltd., Shanghai, China) was injected into the anterior cubital vein with contrast agent injector Missouri-XD2001 (Ulrich GmbH & Co. KG, Ulm, Germany) at a flow rate of 5 mL/s, followed by 40 mL of 0.9% saline solution at the same flow rate. Dynamic scanning began 5 s after the contrast agent was injected, and images were acquired with an exposure time of 0.5 s for each phase and an acquisition interval of 2.5 s. A total of 18 phases were acquired, and the total perfusion time was 56.5 s. A routine whole lung enhancement phase was scanned immediately after CTPI for imaging diagnosis.

### 2.3 Comparison of radiation dose

The CTPI radiation dose indicators for each patient, such as volume CT dosimetry index (CTDI_vol_) and dose length product (DLP), were obtained and recorded from the picture archiving and communication system (PACS). Patients who underwent routine lung enhanced scans (including plain scan, arterial phase, and venous phase) on the same CT during the same period as this study were randomly selected. The number and gender of the patients were consistent with those finally included in the study. The radiation dose of a routine lung-enhanced scan was obtained in the same way, and the radiation dose of CTPI was compared with that of routine lung-enhanced scan and the diagnostic reference level (DRL) of China or the American College of Radiology (ACR) (13).

### 2.4 EGFR detection and grouping

Patients with suspected lung cancer underwent histopathological examinations within 1 week after CTPI, including CT-guided lung puncture, bronchoscopic biopsy, and surgery. Tissue specimens with pathological NSCLC diagnosis were further subjected to immunohistochemical testing (e.g., CD34 staining) and genetic testing. EGFR gene mutation was detected by probe hybridization liquid capture assay and high-throughput sequencing (HTS) technology, including all gene mutation types (e.g., point mutation, indel mutation, copy number mutation, and rearrangement mutation) closely related to the pathogenic mechanism and clinical treatment of lung cancer. According to the EGFR gene mutation status, all patients were divided into the EGFR mutation group and the wild-type group.

### 2.5 Image processing and measurement

All perfusion images were uploaded to the AW Server 4.6 workstation (GE Healthcare, Waukesha, WI, USA) and were then measured using the CT Body Tumor Perfusion 4D software by two radiologists with 6 and 11 years of experience in imaging diagnosis. Circular or oval areas at the layer of the largest cross-sectional area of the target lesion and adjacent upper and lower layers were manually delineated as a region of interest (ROI), while avoiding areas such as large vessels, calcification, liquefaction necrosis, or cavities inside the tumor. The aorta at the same layer of ROI was selected as the reference vessel, and the reference ROI was delineated, avoiding the vessel wall and atherosclerotic plaque. The ROI should be larger than two-thirds of the target lesion or reference vessel area. Finally, the relevant perfusion parameter values, such as, blood flow (BF), blood volume (BV), mean transit time (MTT), time to peak (TTP), time to maximum of the residual function (Tmax), positive enhancement integral (PEI), mean slope of increase (MSI), and permeability surface (PS), were obtained using the software. Both the physicians did not know the pathological results and EGFR mutation status before the measurement. First, the mean value at three layers was calculated by each physician, and then, the two mean values were averaged again as the final perfusion parameter value.

### 2.6 Statistical analysis

Continuous variables were expressed as mean ± standard deviation (x ®±s) or [median (interquartile range) [M (P25, P75)]] for normally distributed and non-normally distributed ones separately. Categorical variables were described as frequencies and percentages. The consistency assessment between two observers was performed with the interclass correlation coefficient (ICC). Numerical differences between the groups were assessed by the *t*-test or the non-parametric test for continuous data and the Chi-square test for categorical variables. Based on significant variables from the univariate analysis, a subsequent multivariate logistic regression analysis was performed to identify independent predictors. Receiver operating characteristic (ROC) curves were constructed, and the area under the curve (AUC) was calculated to assess the predictive performance in terms of sensitivity, specificity, and accuracy. The K-fold cross-validation was performed to evaluate the predictive power of the combined model constructed by logistic regression. The threshold for significance was set at a *p*-value of <0.05. All statistical analyses were conducted using SPSS, Version 22.0 (IBM Corp, IL, USA), MedCalc software, Version 20.014 (Mariakerke, Belgium), and the R Project for Statistical Computing, Version 4.5.1. All assumptions for parametric statistical tests were checked by a professional health statistician.

## 3 Results

### 3.1 Baseline clinical characteristics

A total of 86 patients were finally recruited, including 45 women and 41 men, with an average age of 69 years (ranging from 41 to 90 years). There were 82 (95.3%) adenocarcinoma cases and 4 (4.7%) squamous cell carcinoma cases. In terms of personal history, 32 (37.2%) had a history of smoking and 17 (19.8%) had a family history of malignant tumors. In addition, 82 (95.3%) presented with a solitary lung lesion.

There were 47 cases and 39 cases in the EGFR mutation group and the wild-type group, respectively, with a mutation rate of 54.7%. More women (*p* = 0.065) and non-smokers (*p* = 0.828) were in the EGFR mutation group, but the differences were not significant. In addition, no statistical differences between the two groups were found in age, family history of malignant tumor, and number of lesions, with all *p*-values greater than 0.05. See Table 1 for details.

**Table 1:** Association between clinical characteristics and EGFR mutation among patients with NSCLC.

Characteristic	Total	Mutation group	Wild-type group	Statistic	*p*
Number	86	47 (54.7%)	39 (45.3%)		
Age (years)	69 (64, 74)	70 (63, 76)	69 (65, 73)	0.321^*^	0.748
Gender, *n* (%)				0.368^#^	0.065
Women	45 (52.3%)	26 (55.3%)	19 (48.7%)		
Men	41 (47.7%)	21 (44.7%)	20 (51.3%)		
History of smoking, *n* (%)				0.047^#^	0.828
No	54 (62.8%)	30 (63.8%)	24 (61.5%)		
Yes	32 (37.2%)	17 (36.2%)	15 (38.5%)		
Family history of malignant tumor, *n* (%)				0.854^#^	0.355
No	69 (80.2%)	36 (76.6%)	33 (84.6%)		
Yes	17 (19.8%)	11 (23.4%)	6 (15.4%)		
Number of lesions, *n* (%)				0.036^#^	0.849
Single lesion	82 (95.3%)	45 (95.7%)	37 (94.9%)		
Multiple lesions	4 (4.7%)	2 (4.3%)	2 (5.1%)		

^*^Mann–Whitney test; ^#^Chi-square test.

### 3.2 Radiation dose

Based on the characteristics of the patients finally included in this study, 86 patients (45 women and 41 men) who underwent a routine lung CT enhanced scan were randomly selected at last. The comparison of the radiation dose of CTPI with that of conventional enhanced lung scan and the DRL of China and ACR is shown in Table 2. The results showed that, although the CTDIvol and DLP of CTPI were higher than those of routine lung-enhanced scans, they were still far lower than the DRL of China and ACR.

**Table 2:** Comparison of radiation dose between CTPI and routine lung CT enhanced scan, as well as DRL of China and ACR.

Radiation dose indicator	CTPI	Routine lung enhanced scan	DRL of China (2018)	DRL of ACR (2017)	*p*
CTDI_vol_ (mGy)	28.87 ± 3.48	14.34 ± 2.75	45^*^	36^*^	<0.05
DLP (mGy·cm)	775.47 ± 91.92	512.32 ± 78.55	1,200^*^	1,329^*^	<0.05

^*^Since DRL is a chest plain scan data, the calculation here is three times that of DRL, which is equivalent to the sum of the plain scan, arterial phase and venous phase. CTPI, CT perfusion imaging; DRL, diagnostic reference level; ACR, American College of Radiology; CTDI_vol_, volume CT dosimetry index; DLP, dose length product.

### 3.3 Inter-observer consistency analysis

The inter-observer consistency is robust, with ICC values greater than 0.85 for all CTPI parameters (Table 3), indicating that the two radiologists’ manual ROI delineation for CTPI parameter measurement is reliable and reproducible.

**Table 3:** Inter-observer consistency for the CTPI parameter measurements.

Parameters (unit)	ICC (*n* = 86)	95% CI	Statistic (*F*)	*p*
BF (ml/min/100 g)	0.959	0.936–0.974	25.854	<0.001
BV (ml/100 g)	0.924	0.883–0.950	13.087	<0.001
MTT (s)	0.969	0.952–0.980	31.650	<0.001
TTP (s)	0.905	0.839–0.942	11.686	<0.001
Tmax (s)	0.947	0.919–0.965	18.965	<0.001
PEI	0.896	0.840–0.932	9.577	<0.001
MSI	0.929	0.890–0.954	14.717	<0.001
PS (ml/min/100 g)	0.954	0.929–0.970	21.513	<0.001

ICC, interclass correlation coefficient; BF, blood flow; BV, blood volume; MTT, mean transit time; TTP, time to peak; Tmax, time to maximum of the residual function; PEI, positive enhancement integral; MSI, mean slope of increase; PS, permeability surface.

### 3.4 Pathological features of typical cases in the two groups

**Figure 2:**
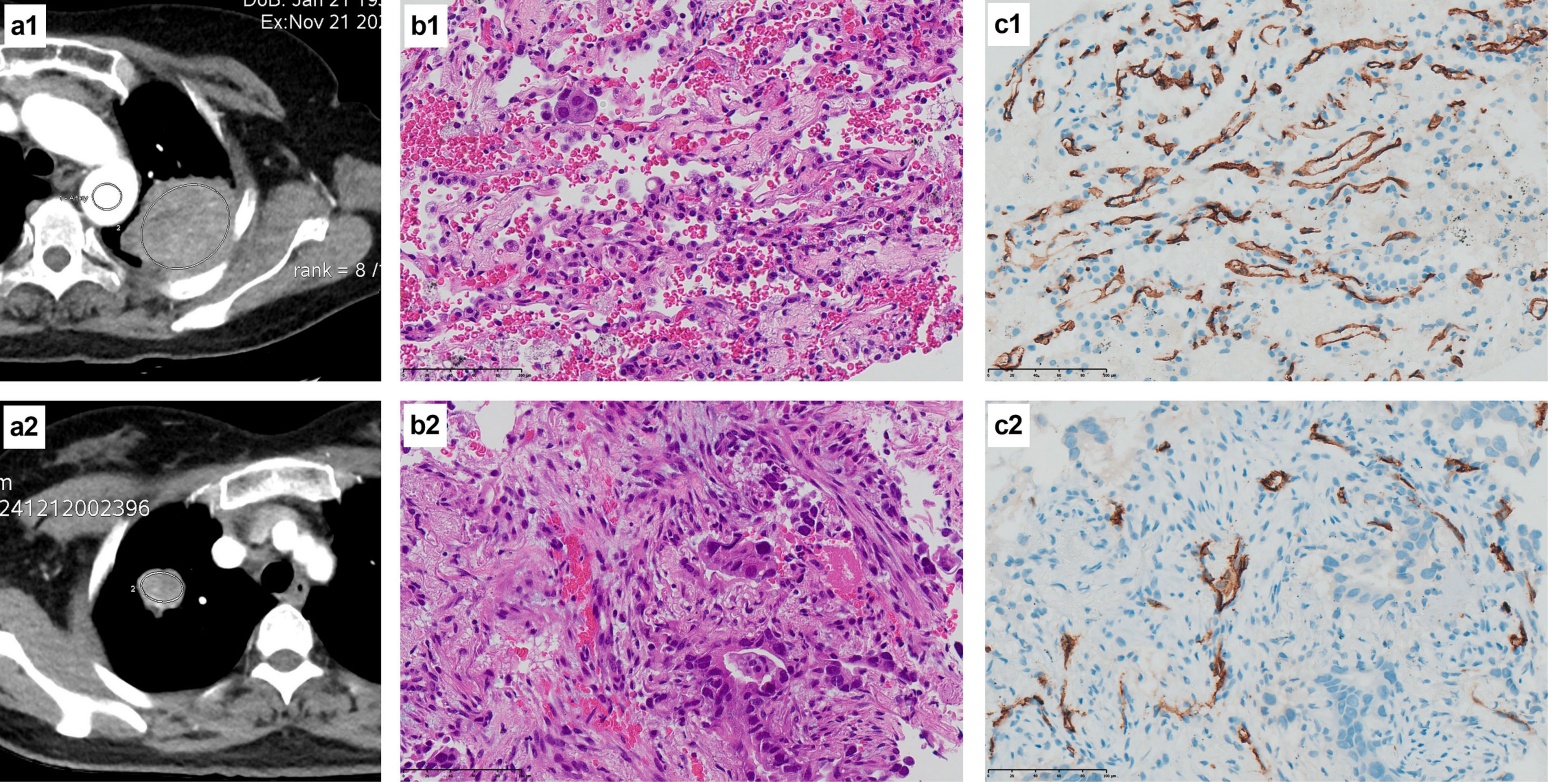
H&E staining and CD34 immunohistochemical staining of the EGFR mutant group and the wild-type group. **(a1)** The CT image of a 53-year-old female patient with a left upper lung mass, and the oval area in the mass is the measured ROI; **(b1)** The pathological diagnosis of this patient was lung adenocarcinoma (H&E staining at ×400 times magnification), and the genetic test result was EGFR mutant type; **(c1)** CD34 immunohistochemical staining of this patient showed a large amount of brownish-yellow positive expression (×400 times magnification), indicating abundant microvessels and neovascularization; **(a2)** The CT image of a 69-year-old female patient with a mass in the right upper lung, with the elliptical area within the mass being the ROI for measurement; **(b2)** The pathological diagnosis of this patient was lung adenocarcinoma (H&E staining at ×400 times magnification), and the genetic test result was EGFR wild type; **(c2)** CD34 immunohistochemical staining of this patient showed a small amount of brownish-yellow positive expression (×400 times magnification), indicating that there were relatively few microvessels and neovascularization.

Tissue specimens from patients with a pathological diagnosis of NSCLC were further subjected to immunohistochemistry, including CD34 staining. CD34 is a marker of vascular endothelial cells that can reflect tumor angiogenesis; the occurrence of brownish-yellow cytoplasm after CD34 staining represents positive expression. Figure 2 indicates that the positive expression of CD34 was significantly more abundant in typical cases from the EFGR mutation group than the wild-type group.

### 3.5 Association between CTPI parameters and EGFR mutation

**Figure 3:**
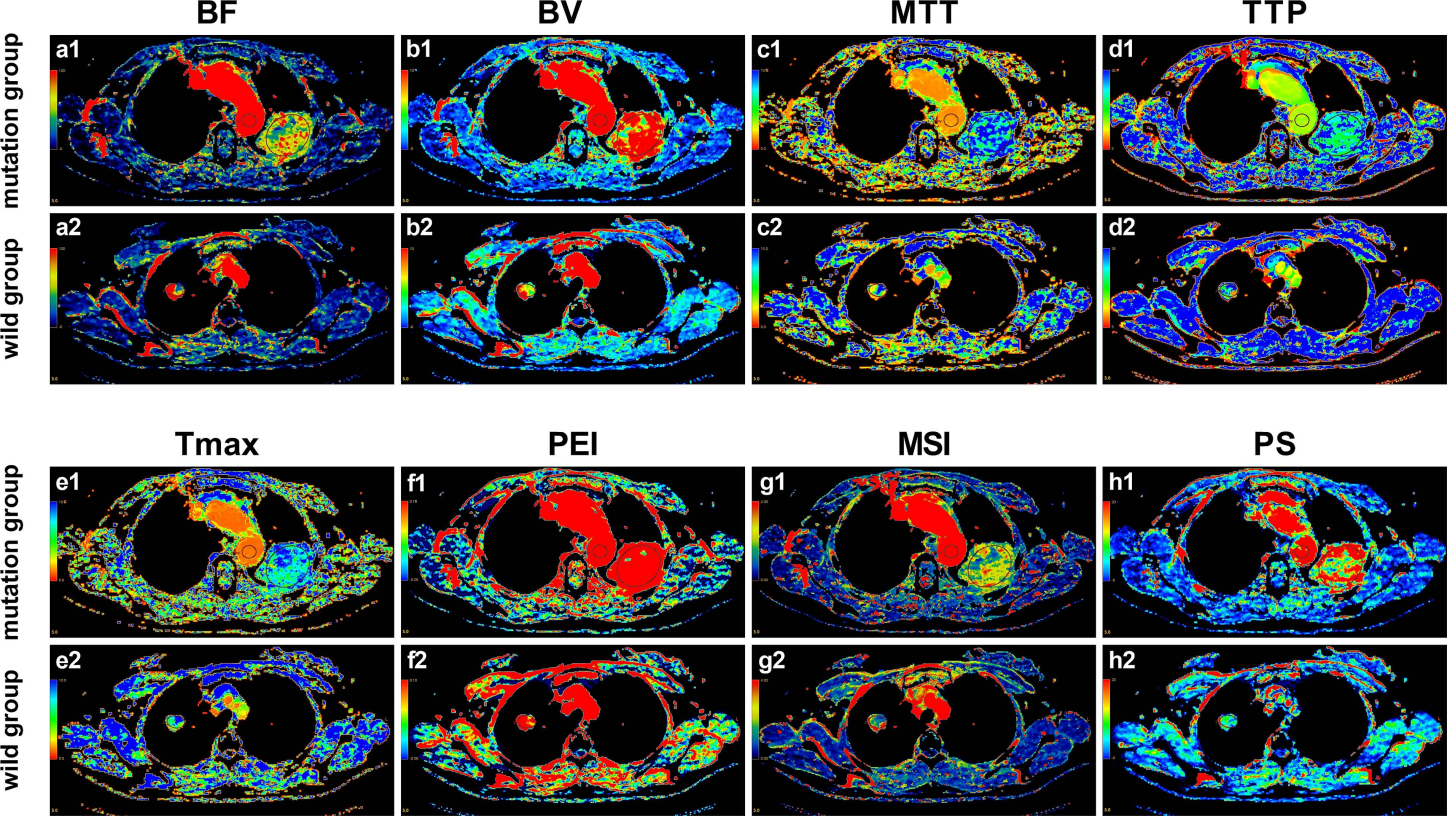
Pseudo-color images of CTPI parameters in the EGFR mutant group and the wild-type group. **(a1–h1)** Pseudo-color images of the perfusion parameters of the 53-year-old female patient with EGFR mutation as mentioned in Figure 2; **(a2–h2)** Pseudo-color images of the perfusion parameters of the 69-year-old female patient with EGFR wild-type as mentioned in Figure 2. It can be observed with the naked eye that, except for Tmax and PEI, there are noticeable differences in the ROI color distributions of the other six parameters between the two groups, especially PS.

Pseudocolor images of CTPI were generated, and the relevant parameters values were measured and obtained using the aforementioned body perfusion analysis software. As shown in Figure 3, except for Tmax and PEI, the ROI color distributions of the other six parameters between the two groups seem to have large differences, especially PS.

**Figure 4:**
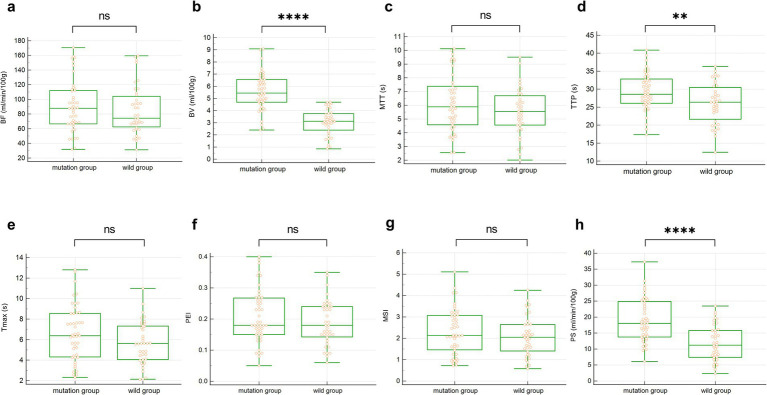
The box plot of univariate analysis between the two groups of CTPI parameters. **(a–h)** BF, BV, MTT, TTP, Tmax, PEI, MSI and PS. ns represents *p* > 0.05, ** represents *p* < 0.01, **** represents *p* < 0.0001.

Furthermore, the univariate analysis in Table 4 showed that, compared with the wild-type group, BV (5.56 ± 1.51 vs. 3.04 ± 1.07, *p* < 0.001), TTP (29.31 ± 5.12 vs. 25.99 ± 5.68, *p* = 0.006), and PS (18.98 ± 6.79 vs. 11.77 ± 5.56, *p* < 0.001) were all higher in the EGFR mutation group. No statistical differences were found in BF, MTT, Tmax, PEI, and MSI, with all *p*-values greater than 0.05. The comparison of all eight quantitative parameters is detailed in Figure 4.

**Table 4:** Association between CTPI parameters and EGFR mutation among patients with NSCLC.

Parameters (unit)	Mutation group (*n* = 47)	Wild-type group (*n* = 39)	Statistic	*p*
BF (ml/min/100 g)	87.88 (66.61, 112.10)	74.35 (62.48, 104.21)	0.664^*^	0.507
BV (ml/100 g)	5.56 ± 1.51	3.04 ± 1.07	9.019^#^	<0.001
MTT (s)	6.16 ± 2.08	5.53 ± 1.69	1.517^#^	0.133
TTP (s)	29.31 ± 5.12	25.99 ± 5.68	2.846^#^	0.006
Tmax (s)	6.58 ± 2.70	5.66 ± 2.09	1.730^#^	0.087
PEI	0.21 ± 0.09	0.19 ± 0.07	1.255^#^	0.213
MSI	2.29 ± 1.07	2.08 ± 0.92	0.972^#^	0.334
PS (ml/min/100 g)	18.98 ± 6.79	11.77 ± 5.56	5.311^#^	<0.001

^*^Mann–Whitney test; ^#^Independent *t*-test. BF, blood flow; BV, blood volume; MTT, mean transit time; TTP, time to peak; Tmax, time to maximum of the residual function; PEI, positive enhancement integral; MSI, mean slope of increase; PS, permeability surface.

### 3.6 Independent predictors and combined predictive model

As shown in Table 5, according to the univariate analysis, we further introduced BV, TTP, and PS into the multivariate logistic regression analysis, and the results identified BV (*p* < 0.001), TTP (*p* = 0.029), and PS (*p* = 0.014) as independent predictors of EGFR mutation.

**Table 5:** Multivariate logistic regression of EGFR mutation based on CTPI parameters.

Parameters (unit)	*β*	S.E.	Wald	OR	95% CI	*p*
BV (ml/100 g)	1.375	0.357	14.860	3.954	(1.966, 7.955)	<0.001
TTP (s)	0.142	0.065	4.796	1.152	(1.015, 1.308)	0.029
PS (ml/min/100 g)	0.162	0.066	5.997	1.176	(1.033, 1.339)	0.014

BV, blood volume; TTP, time to peak; PS, permeability surface.

**Figure 5:**
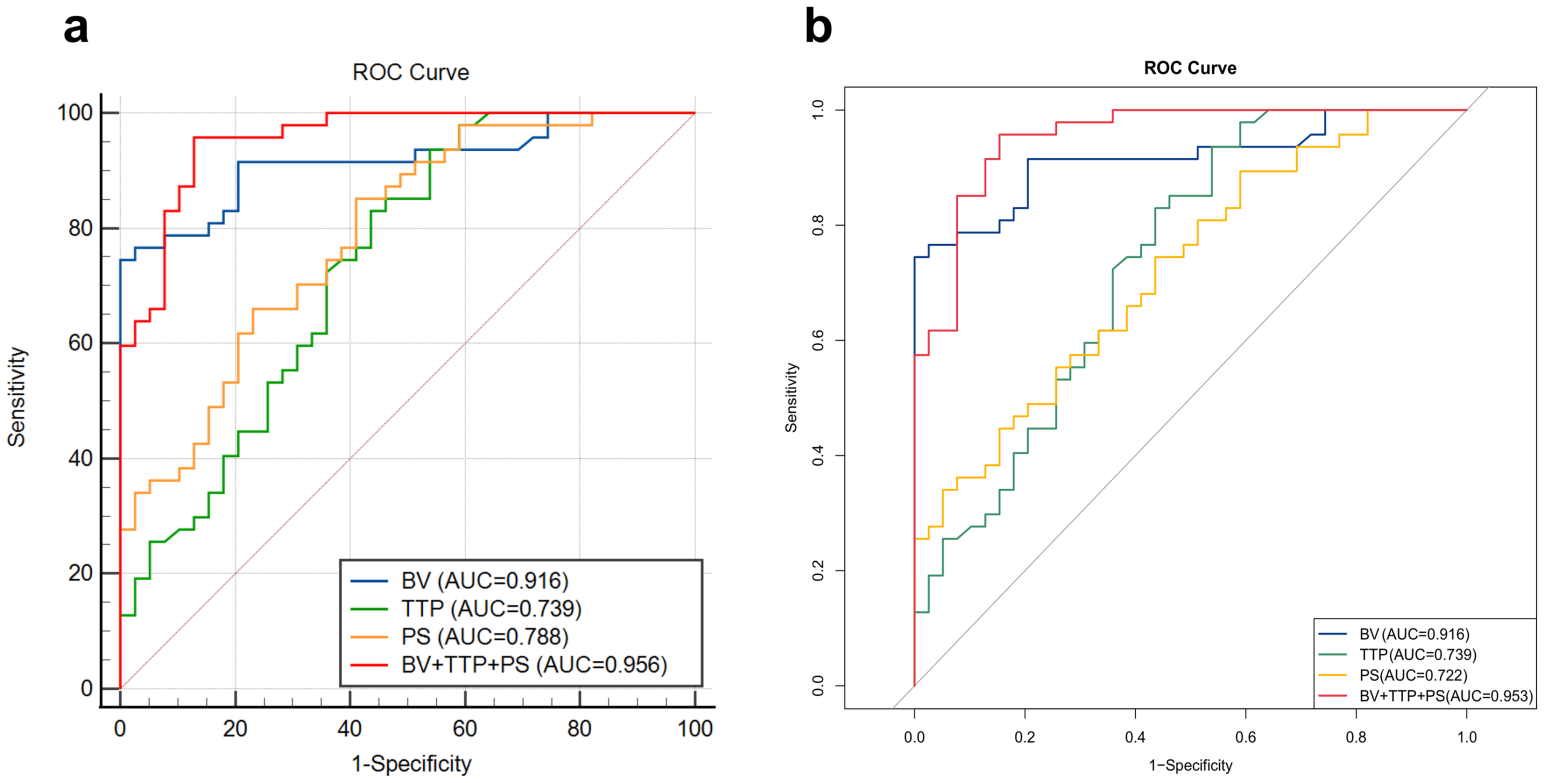
Comparison of ROC curves of independent predictors and the combined prediction model. **(a)** The ability of the combined prediction model constructed by logistic regression and different independent predictors to distinguish EGFR gene mutations. The combined prediction model (BV + TTP + PS) has the highest AUC (0.956), indicating a superior predictive performance. **(b)** K-fold cross-validation shows that the AUC of the combined prediction model is 0.953, indicating that the predictive power of the combined model is stable and reliable.

ROC curves were constructed for the independent predictors screened out. According to ROC, the AUC of BV was 0.916, and its sensitivity, specificity, and accuracy were 74.47, 100, and 82.56%, respectively, with a cut-off value of 4.69. The AUC of TTP was 0.739, and its sensitivity, specificity, and accuracy were 93.62, 46.15 and 67.44%, respectively, with a cut-off value of 23.84. The AUC of PS was 0.788, and its sensitivity, specificity, and accuracy were 85.11, 58.97, and 68.60%, respectively, with a cut-off value of 12.11. The AUC of the combined predictive model (BV + TTP + PS) for EGFR mutation reached 0.956, and its sensitivity, specificity, and accuracy were 95.74, 87.18, and 89.53%, respectively. The predictive performance of the combined model was superior to that of any single parameter (*p* < 0.05). See Table 6 and Figure 5a for details.

**Table 6:** ROC analysis of independent predictors and the combined predictive model for EGFR mutation.

Parameters (unit)	Cut-off value	Sensitivity	Specificity	Accuracy	AUC (95% CI)	*p*
BV (ml/100 g)	4.69	74.47%	100.00%	82.56%	0.916 (0.836–0.965)	<0.05
TTP (s)	23.84	93.62%	46.15%	67.44%	0.739 (0.633–0.828)	
PS (ml/min/100 g)	12.11	85.11%	58.97%	68.60%	0.788 (0.686–0.869)	
BV + TTP + PS	/	95.74%	87.18%	89.53%	0.956 (0.888–0.988)	

BV, blood volume; TTP, time to peak; PS, permeability surface.

K-fold cross-validation was used for internal data validation, and the results showed that the AUC of the combined prediction model was 0.953, which is comparable to the AUC (0.956) of the combined model constructed by the logistic regression analysis performed previously. This finding indicates that the predictive power of the combined model is very stable and reliable. The detailed results of internal validation are shown in Table 7 and Figure 5b.

**Table 7:** Internal validation of independent predictors and the combined predictive model for EGFR mutation.

Parameters (unit)	Cut-off value	Sensitivity	Specificity	Accuracy	AUC (95% CI)	*p*
BV (ml/100 g)	4.69	79.50%	85.11%	82.56%	0.916 (0.854–0.976)	<0.05
TTP (s)	23.84	56.41%	76.60%	67.44%	0.739 (0.632–0.846)	
PS (ml/min/100 g)	12.11	59.00%	68.09%	64.00%	0.722 (0.616–0.828)	
BV + TTP + PS	/	87.18%	89.36%	88.37%	0.953 (0.913–0.993)	

BV, blood volume; TTP, time to peak; PS, permeability surface.

## 4 Discussion

EGFR belongs to the ErbB receptor family of receptor tyrosine kinases (RTKs), and its mutations primarily occur in exons 18–21 of the intracellular tyrosine kinase domain. The two most common subtypes are exon19-Del and exon21-L858R, accounting for a total of 90% of all EGFR mutations (14, 15). Both EGFR 19-Del and 21-L858R are highly sensitive to epidermal growth factor receptor-tyrosine kinase inhibitors (EGFR-TKIs) (16). A series of studies have shown that, compared with standard chemotherapy, the use of EGFR-TKIs significantly improves progression-free survival (PFS) and overall survival (OS) in patients with advanced NSCLC (17, 18) and has become the first-line treatment for patients with EGFR-positive advanced NSCLC.

The occurrence and progression of NSCLC may involve multiple pathogenic factors, with tumor neovascularization as one of the key factors, and EGFR mutation is the primary cause of tumor neovascularization (19). Several studies have shown that the abnormal or elevated expression of EGFR in NSCLC is related to the proliferation, angiogenesis, invasion, metastasis, and inhibition of apoptosis of tumor cells (20, 21). On the one hand, EGFR is activated after binding to its ligand EGF and transforming growth factor-*α* (TGF-α), followed by the change from monomer to dimer and the completion of tyrosine phosphorylation. This dimer can mediate cell signal transduction through the phosphatidylinositol 3-kinase (PI3Ks) pathway, ultimately leading to cell proliferation and angiogenesis (22, 23). On the other hand, the EGFR gene can promote the activation of vascular endothelial growth factor (VEGF) through a hypoxia-independent mechanism, and the more EGFR is expressed, the more obvious is the VEGF activation (24). As a member of the platelet-derived growth factor (PDGF) family, VEGF is currently known to be the most potent factor stimulating angiogenesis and the strongest factor increasing vascular permeability, and it can be expressed in endothelial cells and tumor cells (25). VEGF can directly stimulate receptors to induce endothelial cell proliferation and promote neocapillary formation by binding to receptors on the endothelial cell membrane of pulmonary vessels. At the same time, VEGF can significantly increase vascular permeability through the action of the cellular vesicular apparatus. In addition, the activation of VEGF is able to stimulate the release of VEGFR-3 factor from endothelial cells and promote extracellular matrix degeneration, making it more conducive to tumor vascular growth and distant metastasis (26).

Although histological biopsy is the gold standard for EGFR mutation detection, biopsy still has many limitations in clinical practice. EGFR promotes neovascularization and vascular permeability in NSCLC by mediating PI3Ks signal transduction pathways and inducing increased VEGF expression, which is the pathological basis for its rich internal blood perfusion and rapid flow rate. However, CT perfusion can clearly reflect the blood flow pattern of tissue, and some of its parameters have a significant correlation with tumor neovascularization (27, 28). Therefore, the pathological differences caused by EGFR mutation status between different tumors can theoretically be reflected by the multi-parameter characteristics of CTPI, and this important auxiliary diagnostic value can help the clinical development of individualized treatment plans when biopsy is infeasible. No known research has been conducted to determine EGFR mutation in NSCLC by CTPI quantitative parameters, as well as the construction of a prediction model based on CTPI parameters.

By prospectively analyzing CTPI parameters in 86 patients with NSCLC, our study revealed that BV, TTP, and PS in the EGFR mutation group were significantly higher than those in the wild-type group, which was similar to the principle and conclusion of CT perfusion parameters in distinguishing benign and malignant pulmonary nodules and predicting the prognosis of chemotherapy in patients with NSCLC (29, 30). The multivariate logistic regression analysis showed that BV, TTP, and PS were all independent predictors of EGFR mutations. BV is a parameter that directly reflects BV of the lesion and is determined by the density of the capillary network. Since tumors have high oxygen demand and EGFR mutation increases internal neovascularization, the capillary network density of NSCLC is high with a rich blood supply (31), resulting in elevated BV values. Consistent with Deng et al. (32), who reported that peripheral lung cancer had a higher BV than focal organizing pneumonia, this study proved that the EGFR mutation group possessed a higher BV. The ROC analysis for the assessment of diagnostic performance showed that the AUC of BV was 0.916, suggesting its good predictive power. Using a BV greater than 4.69 mL/100 g as the cut-off value for possible EGFR gene mutation in NSCLC, the sensitivity, specificity, and accuracy were 74.47, 100, and 82.56%, respectively. TTP refers to the time required for the concentration of contrast medium to reach its peak value when passing through the capillaries, and theoretically, as the number of open capillaries increases, blood flow becomes larger, and then, TTP shortens after EGFR mutation. However, this study found that the TTP of the EGFR mutation group was longer than that of the wild-type group, which may be related to the following reasons: first, after EGFR mutation, extensive neovascularization occurred, characterized by intricate luminal configurations and relatively narrow vessel diameters. Second, due to the activation of VEGF, the extracellular matrix undergoes denaturation, which may, to some extent, hinder blood flow within the lumens of small blood vessels. Finally, most lung tumors receive blood supply from both the bronchial artery and the pulmonary artery. The enhancement phase of the bronchial artery typically occurs later than that of the pulmonary artery. In tumors harboring EGFR mutations, the bronchial artery may serve as the predominant source of vascular supply. Based on the possible factors mentioned above, the time to reach its peak for the contrast agent in the tumor increases in the EGFR mutation group. Several studies using TTP to distinguish benign from malignant pulmonary nodules have concluded similarly (33, 34). The AUC of TTP was 0.739, and the cut-off value was 23.84 s, that is, TTP longer than 23.84 s might point out possible EGFR mutation, and the sensitivity, specificity, and accuracy were 93.62, 46.15, and 67.44%, respectively. PS is a parameter that directly reflects capillary permeability in the lesion. EGFR mutations promote the activation of VEGF, which potently increases vascular permeability through the action of small vesicular organelles and provokes the action of VEGFR-3 factors to alter extracellular matrix properties, making it easier for vascular growth and metastasis. The results of this study showed that PS in the EGFR mutation group was significantly higher than that in the wild-type group, which was consistent with a previous study on the predictive ability of PS in different histological subtypes of lung cancer (35). The AUC of PS was 0.788, and when PS is considered to be greater than 12.11 mL/min/100 g as a cut-off value in predicting EGFR, the sensitivity, specificity, and accuracy were 85.11, 58.97, and 68.60%, respectively. According to these results, it could be found that each independent predictor had its own limitations in predicting EGFR mutation, for instance, the sensitivity of BV and the low specificity and accuracy of TTP and PS. As a consequence, we constructed a combined prediction model of BV, TTP, and PS and found that the AUC of the combined predictive model (BV + TTP + PS) for EGFR mutation reached 0.956, and its sensitivity, specificity, and accuracy were 95.74, 87.18, and 89.53%, respectively. The predictive performance of the combined model was superior to that of any single parameter.

Despite the promising outcomes, there are still some limitations in our study. First, this study only conducted internal validation on the 86 patients included, and we did not recruit additional patients for external validation. Otherwise, the predictive power of the combined model would have been assessed more realistically and accurately. Second, the single-center design restricted the study to a southern province of China, which may result in a lack of generalizability. Third, we did not conduct further sub-group analysis for the gene mutation loci (e.g., exon19-Del and exon21-L858R), while standardized subgroup analysis would deepen the understanding of the potential association between different mutation sites and molecular imaging, so as to better assist clinical treatment decisions. Furthermore, we did not compare CTPI with other non-invasive methods, such as liquid biopsy, so this study could not demonstrate the advantages of CTPI over other non-invasive methods in clinical practice. Finally, our study sample size was relatively small. In the future, our research will further incorporate demographic data from diverse regions for multicenter validation, standardize grouping analysis of gene mutation sites, and utilize machine learning techniques for image analysis, with a particular focus on integrating radiomics and AI-based models.

## 5 Conclusion

Among all quantitative parameters of CTPI, BV, TTP, and PS were independent predictors of EGFR mutation in patients with NSCLC. Specifically, BV > 4.69 mL/100 g, or TTP > 23.84 s, or PS > 12.11 mL/min/100 g might point out the occurrence of EGFR mutation. In addition, the CTPI parameters of the combined model (BV + TTP + PS) had the highest predictive performance and could be more reliable than any single parameter. It is expected to be an important auxiliary diagnostic basis in clinical practice for non-invasive prediction of EGFR gene mutation in patients with NSCLC when tissue samples cannot be obtained by biopsy or surgery.

## Data Availability

The original contributions presented in the study are included in the article/supplementary material, further inquiries can be directed to the corresponding author.
